# Mitochondrial Dysfunction: The Silent Catalyst of Kidney Disease Progression

**DOI:** 10.3390/cells14110794

**Published:** 2025-05-28

**Authors:** Nikola Pavlović, Marinela Križanac, Marko Kumrić, Katarina Vukojević, Joško Božić

**Affiliations:** 1 Department of Anatomy, Histology and Embryology, University of Split School of Medicine, 21000 Split, Croatia; nikola.pavlovic@mefst.hr (N.P.); katarina.vukojevic@mefst.hr (K.V.); 2 Department of Paediatrics, University Hospital of Split, 21000 Split, Croatia; makrizanac@kbsplit.hr; 3 Department of Pathophysiology, University of Split School of Medicine, 21000 Split, Croatia; marko.kumric@mefst.hr; 4 Laboratory for Cardiometabolic Research, University of Split School of Medicine, 21000 Split, Croatia; 5 Mediterranean Institute for Life Sciences, University of Split, 21000 Split, Croatia; 6 Center for Translational Research in Biomedicine, University of Split School of Medicine, 21000 Split, Croatia

**Keywords:** mitochondria, renal pathology, chronic kidney disease, acute kidney injury, CAKUT, mitophagy

## Abstract

Mitochondrial dysfunction is a pivotal driver in the pathogenesis of acute kidney injury (AKI), chronic kidney disease (CKD), and congenital anomalies of the kidney and urinary tract (CAKUT). The kidneys, second only to the heart in mitochondrial density, rely on oxidative phosphorylation to meet the high ATP demands of solute reabsorption and filtration. Disrupted mitochondrial dynamics, such as excessive fission mediated by Drp1, exacerbate tubular apoptosis and inflammation in AKI models like ischemia–reperfusion injury. In CKD, persistent mitochondrial dysfunction drives oxidative stress, fibrosis, and metabolic reprogramming, with epigenetic mechanisms (DNA methylation, histone modifications, non-coding RNAs) regulating genes critical for mitochondrial homeostasis, such as *PMPCB* and *TFAM*. Epigenetic dysregulation also impacts mitochondrial–ER crosstalk, influencing calcium signaling and autophagy in renal pathology. Mitophagy, the selective clearance of damaged mitochondria, plays a dual role in kidney disease. While PINK1/Parkin-mediated mitophagy protects against cisplatin-induced AKI by preventing mitochondrial fragmentation and apoptosis, its dysregulation contributes to fibrosis and CKD progression. For instance, macrophage-specific loss of mitophagy regulators like MFN2 amplifies ROS production and fibrotic responses. Conversely, BNIP3/NIX-dependent mitophagy attenuates contrast-induced AKI by suppressing NLRP3 inflammasome activation. In diabetic nephropathy, impaired mitophagy correlates with declining eGFR and interstitial fibrosis, highlighting its diagnostic and therapeutic potential. Emerging therapeutic strategies target mitochondrial dysfunction through antioxidants (e.g., MitoQ, SS-31), mitophagy inducers (e.g., COPT nanoparticles), and mitochondrial transplantation, which mitigates AKI by restoring bioenergetics and modulating inflammatory pathways. Nanotechnology-enhanced drug delivery systems, such as curcumin-loaded nanoparticles, improve renal targeting and reduce oxidative stress. Epigenetic interventions, including PPAR-α agonists and KLF4 modulators, show promise in reversing metabolic reprogramming and fibrosis. These advances underscore mitochondria as central hubs in renal pathophysiology. Tailored interventions—ranging from Drp1 inhibition to mitochondrial transplantation—hold transformative potential to mitigate kidney injury and improve clinical outcomes. Additionally, dietary interventions and novel regulators such as adenogens are emerging as promising strategies to modulate mitochondrial function and attenuate kidney disease progression. Future research should address the gaps in understanding the role of mitophagy in CAKUT and optimize targeted delivery systems for precision therapies.

## 1. Introduction

Mitochondria are double membrane-bound organelles with distinct morphological features that regulate signaling pathways, energy metabolism, cell differentiation, proliferation, and apoptosis [1,2,3,4,5,6,7]. Additionally, mitochondria are essential for energy production and serve as signaling hubs that regulate cellular responses to stress, including oxidative damage and inflammation. More than 40% of mitochondrial proteins are associated with human diseases, underscoring the crucial role of mitochondria in overall health. Mitochondrial diseases can manifest at any age and affect any organ system, either tissue-specific or multisystemic, with various inheritance patterns [8,9,10]. Due to their genetic material, mitochondria differ from nuclear components, and their dysfunction is associated with numerous diseases, including those affecting the kidneys [9].

The kidney is one of the body’s most vital organs, playing a crucial role in maintaining homeostasis within the human body. The kidneys comprise a complex three-dimensional nephron structure that responds to various extracellular, inflammatory, neurological, and hormonal signals [11,12,13]. Kidney diseases are frequently present as syndromes in clinical settings, and they have various pathological classifications. Common symptoms include unusual urination, swelling, and fatigue [14,15,16]. The percentage of deaths attributed to kidney disease has consistently risen over the last twenty years. Kidney dysfunction has now become the seventh leading risk factor for mortality [17,18,19]. One in ten people globally suffers from chronic kidney disease (CKD), which is the tenth leading cause of death worldwide. Diabetic kidney disease (DKD) and hypertensive kidney disease (HKD) account for over 75% of CKD cases. This increasing burden is further intensified by considerable disparities in treatment outcomes, with minority groups and individuals living in poverty facing limited access to renal replacement therapies and experiencing disproportionately poorer health outcomes [20,21,22,23,24].

**Figure 1:**
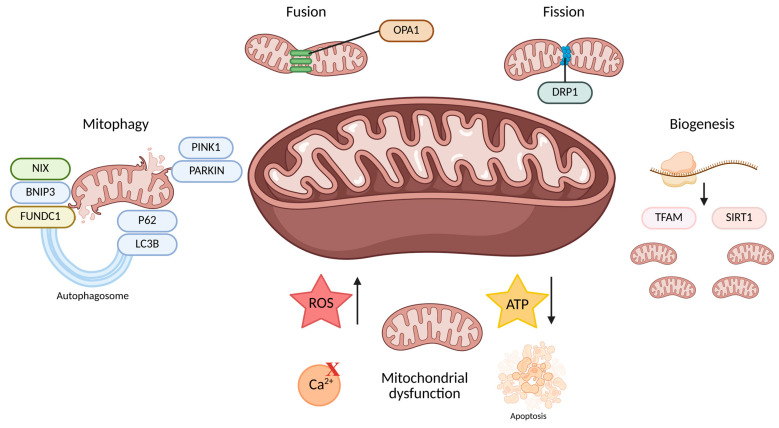
This figure summarizes the mechanisms of mitochondrial quality control, including mitophagy, dynamics (fusion and fission), biogenesis, and the consequences of mitochondrial dysfunction. Fusion, mediated by OPA1, maintains mitochondrial function by merging membranes, while fission, mediated by DRP1, separates damaged mitochondria for removal. Mitophagy selectively eliminates dysfunctional mitochondria through pathways involving PINK1, PARKIN, NIX, BNIP3, FUNDC1, LC3B, and P62. Biogenesis, regulated by TFAM and SIRT1, replenishes mitochondria. Mitochondrial dysfunction leads to increased ROS, disturbed Ca^2^⁺ signaling, decreased ATP production, and activation of apoptosis. Created with Biorender (accessed on 1 May 2025). Abbreviations: NIX, NIP3-like protein X; BNIP3, BCL2/Adenovirus E1B 19 kDa protein-interacting protein 3; FUNDC1, FUN14 domain-containing protein 1; PINK1, PTEN-induced kinase 1; PARKIN, Parkin RBR E3 ubiquitin protein ligase; P62, Sequestosome 1 (also called SQSTM1); LC3B, Microtubule-associated protein 1A/1B-light chain 3B; OPA1, Optic atrophy 1 protein; DRP1, dynamin-related protein 1; TFAM, mitochondrial transcription factor A; SIRT1, Sirtuin 1; ROS, reactive oxygen species; ATP, adenosine triphosphate.

After the heart, the kidneys have one of the highest demands on the body’s mitochondrial content and oxygen consumption. The reabsorption of solutes by the tubule cells is an energy-intensive process that requires a large amount of ATP, primarily generated through oxidative phosphorylation. This results in these cells having the highest mitochondrial content in the kidney [19,25,26]. Mitochondrial dysfunction generates excessive reactive oxygen species (ROS), disturbing redox balance and promoting oxidative stress, contributing to inflammation, fibrosis, and progressive kidney damage (Figure 1) [27]. In parallel, damaged mitochondria release mtDNA and other damage-associated molecular patterns (DAMPs) that trigger immune responses, e.g., via the cGAS-STING pathway, thereby promoting inflammation and immune cell recruitment and contributing to kidney injury and disease progression [26,28]. Furthermore, impaired mitochondrial function drives myofibroblast activation and extracellular matrix accumulation, contributing to tubulointerstitial fibrosis—a key pathological feature in the progression of chronic kidney disease (CKD) [29,30]. In addition, kidney diseases are correlated with impaired mitochondrial homeostasis, which encompasses the dysregulation of mitochondrial biogenesis, fusion dynamics, proteolytic activity, and mitophagy-mediated degradation [31,32].

Given the mitochondria’s central role in kidney health and disease, targeting mitochondria for the treatment of kidney disease is crucial. A recent review by Takasu et al. provides a comprehensive overview of mitochondrial dysfunction in diabetic kidney disease, with a particular emphasis on altered mitochondrial dynamics, oxidative stress, and impaired mitophagy. They also discuss the therapeutic potential of agents such as SGLT2 inhibitors and GLP-1 receptor agonists in restoring mitochondrial function in DKD [33]. While their review focuses specifically on diabetic nephropathy, our article expands this discussion to encompass a broader range of kidney diseases, including AKI, CKD, and CAKUT anomalies, and further explores emerging mechanisms such as epigenetic regulation and mitochondrial-ER crosstalk. This broader perspective underscores the centrality of mitochondria in diverse renal pathologies and highlights additional therapeutic avenues.

## 2. Mitochondria in Kidney Health and Disease

The mitochondrion is a double-membrane organelle rich in enzymes, porins, and translocases in the outer mitochondrial membrane (OMM). These molecules are essential for exchanging metabolites and cations with the cytosol. In addition, apoptosis is closely linked to the OMM, as pro-apoptotic factors can be released from the intermembrane space (space between the outer and inner mitochondrial membranes) when the OMM is excessively permeabilized. In the process of apoptosis, the permeability of the OMM to a variety of pro-apoptotic proteins increases, releasing the lethal proteins into the cytoplasmic matrix and promoting apoptosis. The inner mitochondrial membrane (IMM) contains highly impermeable lipid patterns, such as the phospholipid cardiolipin. There are more proteins in the IMM (about one-fifth of all proteins contained in mitochondria) than in the OMM. Hence, the IMM is responsible for complex biochemical reactions, including transporting glutamic acid, ornithine, and nucleotides, oxidative phosphorylation, ATP synthesis, and regulating mitochondrial fission and fusion [34,35].

Mitochondrial dynamics, i.e., the balance between fusion and fission of mitochondria, is a tightly regulated process that plays a crucial role in maintaining cellular homeostasis. This dynamic system is essential for key physiological functions such as cell cycle progression, programmed cell death, and the maintenance of mitochondrial integrity. Under normal conditions, fusion and fission are well coordinated and ensure proper mitochondrial form and function. However, this balance often shifts towards excessive fission in pathological conditions, leading to mitochondrial fragmentation and subsequent cell damage. A key result of disrupted mitochondrial dynamics is the excessive generation of reactive oxygen species (ROS), which, when sustained, can lead to oxidative harm to proteins, lipids, and DNA, thereby playing a significant role in advancing kidney disease [36,37,38,39,40,41].

The kidneys receive around 20% of the heart’s output, processing roughly 180 liters of glomerular filtrate daily. Due to their role in waste removal, nutrient reabsorption, fluid and electrolyte balance, acid–base regulation, and blood pressure control, kidneys have a high metabolic demand and are rich in mitochondria. Mitochondria play a central role in kidney function, reflecting the organ’s high metabolic demands [42,43]. The kidney ranks just behind the heart in mitochondrial density and oxygen consumption, as large amounts of ATP are required to fuel active transport processes essential for filtration, reabsorption, and secretion. The renal cortex, densely packed with proximal and distal tubules, is particularly rich in mitochondria due to its role in reabsorbing water, ions, and nutrients. Proximal tubular cells, which perform most of this task, rely heavily on mitochondrial energy and must dynamically adjust to fluctuations in energy availability to maintain metabolic efficiency. In contrast, fewer mitochondria are present in the collecting duct cells and specific segments of the Henle loop. However, intercalated cells in the collecting duct still depend on mitochondrial activity for acid–base and electrolyte balance. In addition to ATP production, renal mitochondria are involved in apoptosis regulation, calcium and iron homeostasis, and steroid synthesis, underscoring their critical role in maintaining cellular and organ-level homeostasis [42,43,44,45,46,47].

While much of the focus on mitochondrial dysfunction in kidney disease has centered on tubular epithelial cells, emerging evidence highlights the critical role of mitochondria in glomerular podocytes, which are essential for maintaining the filtration barrier. Podocytes have high energy demands and rely heavily on mitochondrial ATP production to sustain their complex cytoskeletal architecture and slit diaphragm integrity. Disruptions in mitochondrial dynamics, including altered fusion and fission processes, as well as impaired mitophagy, contribute to podocyte injury, leading to proteinuria and glomerulosclerosis—hallmarks of chronic kidney disease (CKD) [48,49]. Moreover, mitochondrial damage can lead to the release of mitochondrial DNA (mtDNA) into the cytosol and extracellular space, acting as a damage-associated molecular pattern (DAMP). The recognition of mtDNA by innate immune receptors, such as Toll-like receptor 9 (TLR9) and the cGAS-STING pathway, triggers sterile inflammatory responses in podocytes and tubular cells, thereby exacerbating renal injury and fibrosis. This mtDNA-driven inflammation highlights a crucial connection between mitochondrial dysfunction and immune activation in the pathogenesis of kidney disease. Incorporating these insights broadens our understanding of mitochondrial contributions beyond tubular segments and highlights potential therapeutic targets for preserving mitochondrial integrity and modulating mtDNA-induced inflammation [50,51,52,53,54].

Mitochondrial dysfunction is closely associated with several kidney diseases, including acute kidney injury (AKI) and chronic kidney disease (CKD) [48,49]. AKI can occur due to ischemic, toxic, or inflammatory insults that impair mitochondrial energy production, increase oxidative stress, and lead to the apoptosis or necrosis of tubular cells. Disrupted mitochondrial dynamics, characterized by imbalances in fission and fusion and defective mitophagy, worsen AKI by allowing damaged mitochondria to accumulate, further intensifying oxidative damage and inflammation. In CKD, ongoing mitochondrial dysfunction contributes to renal fibrosis through metabolic reprogramming, persistent oxidative stress, and damage to mitochondrial DNA (mtDNA), particularly in diabetes and IgA nephropathy [19,26,55,56].

Emerging evidence also suggests that mitochondrial dysfunction plays a role in congenital anomalies of the kidney and urinary tract (CAKUT). Genetic mutations that affect mitochondrial proteins or metabolic pathways can disrupt nephrogenesis, leading to structural malformations such as hypoplastic kidneys or urinary tract obstructions. For instance, mutations in genes related to coenzyme Q10 biosynthesis or components of the mitochondrial electron transport chain have been linked to tubular dysgenesis and cystic kidney phenotypes, emphasizing the importance of mitochondrial integrity in early kidney development [57,58,59].

**Figure 2:**
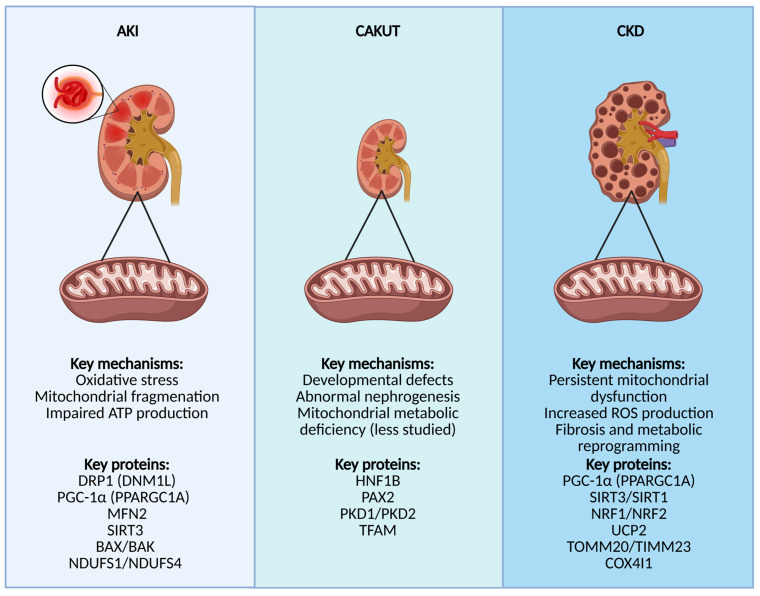
This figure highlights mitochondrial dysfunction in different kidney diseases: AKI, CAKUT, and CKD. In AKI, oxidative stress, mitochondrial fragmentation, and impaired ATP production are key pathological features. In CAKUT, developmental defects and abnormal nephrogenesis are associated with mitochondrial metabolic deficiencies. In CKD, persistent mitochondrial dysfunction, increased ROS production, fibrosis, and metabolic reprogramming drive disease progression. Key regulatory proteins involved in each condition are also indicated. Created with Biorender (Accessed on 1 May 2025). Abbreviations: AKI, acute kidney injury; DRP1 (DNM1L), Dynamin-1-like protein; PGC-1α (PPARGC1A), peroxisome proliferator-activated receptor gamma coactivator 1-alpha; MFN2, Mitofusin-2; SIRT3, Sirtuin-3; BAX, Bcl-2-associated X protein; BAK, Bcl-2 homologous antagonist/killer; NDUFS1/NDUFS4, NADH:ubiquinone oxidoreductase core subunit S1/S4; CAKUT, congenital anomalies of the kidney and urinary tract; HNF1B, Hepatocyte nuclear factor 1-beta; PAX2, Paired box gene 2; PKD1/PKD2, polycystic kidney disease 1/2 proteins; TFAM, mitochondrial transcription factor A; CKD, chronic kidney disease; SIRT1, Sirtuin-1; NRF1/NRF2, Nuclear respiratory factor 1/2; UCP2, Uncoupling protein 2; TOMM20/TIMM23, Translocase of outer mitochondrial membrane 20/Translocase of inner mitochondrial membrane 23; COX4I1, Cytochrome c oxidase subunit 4 isoform 1.

Mitochondrial dysfunction is increasingly recognized as a key factor in the pathogenesis and progression of acute kidney injury (AKI) and chronic kidney disease (CKD) (Figure 2).

### 2.1. AKI

Acute kidney injury (AKI), formerly known as acute renal failure, is a sudden decline in kidney function that develops within hours to days and results in the kidney’s inability to filter waste products and maintain the body’s fluid, electrolyte, and acid–base balance. AKI can range from the mild impairment of kidney function to complete kidney failure and is often triggered by another serious illness, surgery, infection, or certain medications rather than by direct physical injury to the kidneys. The leading causes of AKI are classified as pre-renal (reduced blood flow to the kidneys), intrinsic (direct damage to kidney tissue), and post-renal (obstruction of urine flow due to conditions such as kidney stones or an enlarged prostate) [60,61,62,63,64,65].

Diagnosis usually involves blood tests to measure creatinine and urea levels, urine tests, and imaging tests such as ultrasound or CT scans to determine the underlying cause and assess the extent of kidney damage. In some cases, a kidney biopsy may be performed [66,67].

### 2.2. CKD

In chronic kidney disease (CKD), the persistent dysregulation of mitochondrial homeostasis, including defects in mitochondrial structure, dynamics, and biogenesis, results in impaired energy metabolism, the increased production of reactive oxygen species (ROS), and heightened oxidative stress. These factors contribute to renal inflammation, tubular atrophy, and interstitial fibrosis. Reduced mitochondrial function in renal tubular cells, particularly the shift from oxidative phosphorylation to glycolysis, has been linked to tubular injury and fibrosis, the primary features of CKD progression. In addition, changes in the shape and structure of mitochondria are closely related to their loss of function, further exacerbating kidney damage [68,69].

The diagnosis of CKD typically involves blood tests to measure creatinine and calculate the estimated glomerular filtration rate (eGFR), which assesses how well the kidneys filter waste from the blood. Urine tests are also used to check for the presence of protein or blood, which can indicate kidney damage. Imaging studies such as ultrasound may be performed to evaluate the size and structure of the kidneys and rule out other causes of kidney dysfunction. Sometimes, a kidney biopsy may be necessary to determine the underlying cause and extent of kidney damage. Early detection and regular monitoring are crucial for managing CKD and slowing its progression [70,71,72].

### 2.3. CAKUT

Additionally, mitochondrial dysfunction has been identified as a crucial factor in the pathogenesis of congenital anomalies of the kidney and urinary tract (CAKUT). CAKUT encompasses a broad spectrum of developmental malformations of the kidneys and urinary tract that result from impaired embryonic development. CAKUT is characterized by developmental defects and abnormal nephrogenesis related to mitochondrial metabolic deficiencies, involving proteins such as HNF1B, PAX2, PKD1/PKD2, and TFAM, although this area is less well studied. These mitochondrial abnormalities can disrupt critical developmental pathways, including branching morphogenesis and nephron formation, leading to structural abnormalities such as renal agenesis or hypoplasia/dysplasia. Understanding the role of mitochondrial defects in CAKUT provides insight into disease mechanisms. It opens potential avenues for targeted therapies to restore mitochondrial health to prevent or attenuate congenital renal malformations [73,74,75].

## 3. How Does Epigenetics Shape Mitochondrial Function in Renal Pathology?

Epigenetic mechanisms, including DNA methylation, post-translational modifications of histone proteins, and non-coding RNA (ncRNA) regulations, significantly influence mitochondrial function in renal pathology by regulating genes involved in mitochondrial dynamics, oxidative stress responses, and organelle interactions [76].

DNA methylation can alter the expression of key mitochondrial genes such as PMPCB, AU RNA binding protein/enoyl-CoA hydratase, and TSFM, which are involved in mitochondrial protein processing, translation, and elongation. This can impact mitochondrial morphology, energy production, and ROS generation [40]. Changes in DNA methylation patterns can either upregulate or suppress these genes, influencing mitochondrial dynamics and function in kidney cells.

Histone modifications, such as acetylation and methylation, further regulate mitochondrial biogenesis and fission–fusion processes. For example, the acetylation of mitochondrial transcription factor A (TFAM) by GCN5L1 impairs its translocation to mitochondria, reducing mtDNA replication and transcription and exacerbating kidney injury [77]. Additionally, increased histone H3K27 acetylation and phosphorylation of Drp1 at serine 616 (p-Drp1S616) promote mitochondrial fission and fibroblast activation, leading to fibrosis and oxidative stress in diabetic nephropathy [78]. Conversely, phosphorylation of Drp1 at serine 637 (p-Drp1S637) inhibits fission, favoring mitochondrial elongation and longevity. Histone methylation also influences mitochondrial gene expression; for instance, EZH2-mediated H3K27 methylation contributes to tubular damage and mitochondrial dysfunction, while mitochondrial S-adenosylmethionine (SAM) levels regulate histone methyltransferases involved in these processes [79,80].

Furthermore, ncRNAs, such as microRNAs (miRNAs), modulate mitochondrial dynamics by targeting transcripts like MTP18 and PPAR-α, affecting mitochondrial fission and fatty acid oxidation, respectively. For example, miR-668 inhibits MTP18 to prevent excessive mitochondrial fission and protect tubular cells from apoptosis during ischemic injury [81,82], whereas miR-17 downregulates PPAR-α, impairing mitochondrial energy metabolism and promoting cyst formation in polycystic kidney disease [83].

Collectively, these epigenetic modifications disrupt mitochondrial homeostasis, leading to increased oxidative stress, impaired bioenergetics, and progression of kidney disease. This highlights their potential as therapeutic targets.

## 4. Mitophagy in Kidney Disease

Autophagy is present in all body tissues as a means of discarding old organelles through lysosomal degradation and recycling of cellular components [84]. However, a special form of autophagy that specifically targets mitochondria is known as mitophagy. Mitophagy occurs either when mitochondrial damage exceeds the cell’s reparatory mechanisms or as a controlled process of maintaining cellular metabolic homeostasis [85]. The first mention of mitophagy dates back to 2008. In a study focusing on the ubiquitin ligase Parkin, known for its role in Parkinson’s disease, researchers discovered that Parkin is selectively recruited to dysfunctional mitochondria with low membrane potential in mammalian cells, thereby triggering the engulfment of autophagosomes [86]. A previous review article portrays a detailed summary of the known signaling pathways of mitophagy and their effector molecules [87]. Briefly, there are two signaling cascades which lead to mitophagy, namely serine/threonine PTEN-induced putative kinase 1 (PINK1)/Parkin-mediated mitophagy and PINK1/Parkin-independent mitophagy through [87]. In the former, PINK1 accumulates on depolarized mitochondria, recruits Parkin to ubiquitinate outer membrane proteins, and signals autophagosome recruitment via adaptors like OPTN/NDP52 that bind LC3. In the latter, the primary effectors are E3 ubiquitin ligases (e.g., ARIH1, MUL1) and autophagy receptors (e.g., BNIP3, FUNDC1, PHB2), which directly recruit autophagosomes, often bypassing ubiquitination [87].

Given the previously mentioned implication of mitochondria in kidney disease, the process of mitophagy in this context should be discussed. When talking about acute kidney injury (AKI), several studies on sepsis, cisplatin toxicity, and ischemia–reperfusion injury models have observed the protective effect of mitophagy in kidney disease [55]. For instance, research on cisplatin, a widely used chemotherapeutic drug with notorious toxicity in the kidneys, showed that PINK1/Parkin-mediated mitophagy is activated in cisplatin nephrotoxicity to protect against kidney injury. The results of one study show that *PINK1* or *Parkin* gene knockout mice, compared to wild-type littermates, show more severe renal functional loss, tissue damage, and apoptosis during cisplatin treatment [87]. Interestingly, the level of mitophagy was reduced in the knockout mice even during basal conditions, without cisplatin treatment. Furthermore, a study published in 2024 discovered a novel protein of interest in mitophagy-related kidney disease research. It presented macrophage migration inhibitory factor (MIF) as a suppressor of mitophagy through the disruption of PINK1–Parkin protein interactions in sepsis-associated acute kidney injury, thereby promoting renal damage. This was in line with previous research that reported increased serum levels of MIF in SA-AKI [88]. In a study by Lin and coworkers, which investigated the pathogenic mechanism of contrast-induced acute kidney injury (CI-AKI), NLR family pyrin domain-containing 3 (NLRP3)-driven inflammation came into focus. It was proven on a mouse model that the inhibition of NLRP3 inflammasome attenuates apoptosis in CI-AKI through the upregulation of Hypoxia-inducible factor 1-alpha (HIF1A) and BCL2 Interacting Protein 3 (BNIP3)-mediated mitophagy [88].

On the other hand, dysregulated mitophagy correlates with disrupted mitochondrial dynamics in AKI and contributes to the development and progression of acute to chronic kidney disease (CKD). Most recent clinical studies on CKD have shown that mitochondrial function decline is associated with the degree of kidney fibrosis in CKD [89]. In line with this, in patients with diabetic nephropathy (DN), urinary mtDNA, which is an indirect indicator of impaired mitophagy, correlated inversely with the estimated glomerular filtration rate (eGFR) and positively with interstitial fibrosis [90]. Furthermore, Cui and colleagues found that the Mitofusin 2 (MFN2) downregulation in macrophages enhances ROS and inhibits mitophagy, driving renal fibrosis [91]. Interestingly, no studies have investigated the influence of mitophagy on CAKUT pathophysiology so far. An indirect implication of the autophagy process has been observed in hypoplastic kidneys (HYP), where a significantly smaller proportion of autophagy marker-positive cells, specifically immunoglobulin heavy chain binding protein (BiP)-positive cells, was found [92]. The PI3K-AKT signaling pathway has also been implicated in CAKUT development through integrative multi-omics data analysis [93]. Since this pathway also regulates mitophagy, a potential mechanistic link between mitophagy and CAKUT warrants exploration.

Mitochondrial dysfunction drives both AKI and CKD through distinct mechanisms. Both conditions feature oxidative stress from ROS overproduction, but mitophagy plays divergent roles—protective in AKI vs. maladaptive in CKD. Unresolved AKI-related mitochondrial damage promotes transition to CKD through persistent inflammation and fibrotic signaling. In summary, mitophagy is a double-edged sword in the context of kidney disease. More specifically, its precise regulation is essential for preventing mitochondrial damage-driven kidney injury, while its dysfunction accelerates disease progression.

## 5. Feeding the Mitochondria: Can Diet Slow Kidney Disease?

Obesity and metabolic syndrome dramatically raise the risk of diabetic nephropathy and chronic kidney disease (CKD) [94,95,96]. A diet designed explicitly for the kidney can promote health and slow its progression to failure [95]. Although mitochondrial dysfunction is a well-known pathway in obesity-related organ damage, its specific impact on the kidney is unclear.

The kidney responds early to a high-fat diet (HFD) in mice, showing increased body weight, reduced plasma adiponectin, and early renal inflammation, including elevated MCP-1 and urinary H₂O₂ levels, observed after just one week of exposure. This initial inflammation precedes albuminuria and may drive obesity-related kidney damage. HFD exerts a biphasic effect on renal mitochondrial function [96,97,98,99]. In the short term, HFD may induce an adaptive response characterized by enhanced mitochondrial antioxidant defenses, such as increased manganese superoxide dismutase (MnSOD) activity and preserved respiratory capacity despite elevated oxidative stress. Upregulated redox-regulating pathways to maintain cellular homeostasis primarily drive this temporary resilience [94,100]. However, these compensatory mechanisms wane with prolonged HFD exposure, typically observed after 16 weeks in rodent models. Mitochondrial copy number declines, biogenesis is impaired due to suppressed PGC-1α expression, and ATP production is significantly reduced. Mitochondrial dysfunction worsens over time, resulting in excessive leakage of reactive oxygen species (ROS), lipid peroxidation, and a heightened vulnerability to further injuries, such as ischemia–reperfusion injury. These maladaptive changes eventually lead to renal fibrosis, glomerular hypertrophy, and damage to tubular epithelial cells, ultimately speeding up the progression of kidney disease [94,100,101,102].

Dietary interventions such as protein and salt restriction are crucial in reducing systemic stressors affecting mitochondrial function and preserving kidney health. High protein intake can elevate intraglomerular pressure, leading to glomerular damage. Simultaneously, a low-protein diet (LPD) of 0.6–0.8 g/kg/day has been shown to reduce proteinuria, slow CKD progression, and improve metabolic acidosis, supporting mitochondrial function [103,104,105,106]. Combining LPD with renin–angiotensin system (RAAS) blockade provides additional renal protection [107,108]. Salt restriction, particularly lowering sodium intake to less than 2,000 mg/day, has been linked to reduced blood pressure and decreased proteinuria, both important for slowing CKD progression. However, the long-term effects of strict salt restriction remain debated, with some studies raising concerns over potential risks such as hypotension in advanced CKD [109,110,111,112]. Key nutrients like coenzyme Q10 (CoQ10) and B vitamins (riboflavin and thiamine) support mitochondrial health and energy production. CoQ10, as an antioxidant, may diminish oxidative stress and enhance renal function, while B vitamins help alleviate energy deficits caused by mitochondrial dysfunction in CKD [113]. Antioxidant-rich foods, such as berries, red bell peppers, and leafy greens, also contribute to this protection by neutralizing reactive oxygen species (ROS), thereby further shielding kidney mitochondria from damage [114].

## 6. Hormonal Guardians of the Mitochondria: Estrogen and Thyroid Hormones in Kidney Disease Regulation

Hormones, particularly estrogen and thyroid hormones, are vital in mitochondrial function and kidney health. Estrogen is vital in regulating mitochondrial dynamics and oxidative stress in kidney disease through various mechanisms. It inhibits the production of ROS by blocking angiotensin II receptor type 1 (AT1R) and mineralocorticoid receptor (MR) signaling, which are typically linked to increased NADPH oxidase activity and intracellular ROS generation. This inhibition helps to reduce oxidative damage in vascular smooth muscle cells (VSMCs) and endothelial cells (ECs), thereby improving kidney function. Additionally, estrogen enhances the expression of antioxidant enzymes, such as superoxide dismutase (SOD), which further reduces oxidative stress [115,116,117,118].

Furthermore, estrogen protects against mitochondrial dysfunction by regulating calcium-induced permeability transition and promoting mitochondrial calcium sequestration through L-type calcium channels. This regulation activates protective signaling pathways, including Src/ERK/CREB via PI3K activation, which helps maintain mitochondrial integrity and prevent apoptosis [119]. By increasing the expression of Bcl-2 family proteins, estrogen inhibits the mitochondrial apoptotic pathway responsible for triggering excessive ROS and calcium concentrations. Overall, these effects preserve mitochondrial function and prevent cell death, underscoring the potential of estrogen as a therapeutic agent in treating kidney disease [120,121,122,123,124,125].

In addition to estrogen, other hormones have been shown to modulate mitochondrial function and influence the progression of kidney disease. Among these, thyroid hormones (T3 and T4) also play a critical yet dual role in mitochondrial function and kidney disease pathophysiology. T3 enhances mitochondrial ATP production and oxygen consumption through mechanisms such as mitochondrial uncoupling proteins (e.g., UCP-2), promoting energy metabolism and increasing renal oxygen demand, which may induce cortical hypoxia and exacerbate nephropathy [126,127,128]. Conversely, hypothyroidism, which is common in CKD patients, is associated with reduced mitochondrial efficiency, oxidative stress, and accelerated kidney dysfunction. Thyroid hormones modulate antioxidant defenses, such as superoxide dismutase (SOD), and interact with the renin–angiotensin–aldosterone system (RAAS), further influencing oxidative stress, and fibrosis. These findings highlight the delicate balance of thyroid hormone signaling in mitochondrial homeostasis and kidney health, underscoring their potential as therapeutic targets in CKD management [126,127,128,129,130,131,132].

## 7. Therapeutic Approaches for Kidney Disease

Mitochondria-targeted therapies for kidney diseases have shown promise in addressing mitochondrial dysfunction, particularly in reducing oxidative stress, improving mitochondrial dynamics, and enhancing biogenesis. However, current evidence largely stems from preclinical studies, with limited translation to clinical practice despite some advances.

Several studies assessed the effect of mitophagy as a therapy target for acute and chronic kidney injury. A study conducted on ischemic AKI in mouse models and gentamicin-induced AKI in the zebrafish model demonstrated that cobaltosilicate oxide-polyethylene glycol-triphenylphosphine (COPT) nanoparticles ameliorate the transition from acute to chronic kidney disease by inducing BNIP3-mediated mitophagy [133]. Additionally, paeoniflorin (PF), a water-soluble monoterpene glycoside extracted from *Paeonia lactiflora*, suppresses kidney inflammation by regulating mitophagy [134]. The mechanism through which it exerted its beneficial effect was by promoting macrophage polarization from M1 to M2 and inducing mitophagy via regulating Krüppel-like transcription factor 4 (KLF4) and upregulating mitophagy-related proteins PINK1, Parkin, Bnip3, P62, and LC3 in vivo and in vitro. In line with this, a study dealing with immune-regulatory effects on macrophages observed the ability of rapid-releasing hydrogen sulfide (H2S) donor NaSH, and a slow-releasing H2S compound S-propargyl-cysteine (SPRC) to protect the heart and kidney from tissue injury induced by LPS [135]. The study again portrayed a link between macrophage polarization from M1 to M2 and the PINK1/Parkin-mediated mitophagy pathway. Furthermore, treatment with the peroxisome proliferator-activated receptor-α (PPAR-α) agonist could reduce the pathology of polycystic kidney disease (PKD) and potentially improve the renal function of the disease by modulating mitophagy [136]. Additional information about targeting mitophagy or mitophagy-related pathways as a treatment for kidney disease can be found in Table 1.

Furthermore, an interesting approach to treating renal tubular injury using mitochondria transplantation (MITO) was employed [150].The study found that MITO, a process in which exogenous isolated mitochondria are taken up by cells, can mitigate AKI both in vitro and ex vivo. The molecular basis included the modulation of genes and pathways most consistent with mitochondrial biogenesis and energy metabolism, thereby reducing kidney damage. Additionally, RNAseq detected the downregulation of genes involved in neutrophil recruitment, including IL1A, CXCL8, and PIK3R1. Diabetes mellitus (DM) often leads to an increase in oxidative stress, which contributes to the development of diabetes complications, including diabetic kidney disease (DKD) [150]. One study, which investigated the effects of ethyl acetate extract of *Potentilla indica* on streptozotocin-induced diabetic male rats, found a protective effect [150]. The study emphasizes the importance of phenolic compounds in Potentilla indica extract for its renoprotective effect. Through their potent antioxidant activity, these compounds reduce ROS production, lipid peroxidation, and improve mitochondrial respiratory chain complex activity, as well as glutathione peroxidase (GSH-Px), superoxide dismutase (SOD), and catalase (CAT) activities. In line with another study that dealt with obesity-linked DN, a positive effect of L-carnitine was found to be exerted through improvements in ROS production and SOD expression in the kidney, among other effects [151]. Furthermore, Hallows and coworkers found that bempedoic acid treatment in hypercholesterolemia might benefit polycystic kidney disease [152]. Specifically, in Polycystic Kidney Disease 1 (Pkd1)-null kidney cells and ATP Citrate Lyase (Acly) knockdown cells, BA inhibited mitochondrial superoxide production and promoted mitochondrial elongation, suggesting improved mitochondrial function. Although several studies have shed light on the role of Pyruvate kinase M2 (PKM2) in mitochondrial regulation, one study discovered that PKM2 binds to myosin heavy chain 9 (MYH9) to promote dynamin-related protein 1 (DRP1)-mediated mitochondrial fragmentation [153]. According to the study, in the case of staurosporine or cisplatin AKI, the regulation of PKM2 activity partially limits mitochondrial fragmentation, thereby directly decreasing the level of renal tubular injury and cell death, including apoptosis, necroptosis, and ferroptosis. Yan and colleagues reported an advanced technique for treating AKI in 2023. They studied tetrahedral framework nucleic acid (tFNA) as a vehicle and combined typhaneoside (Typ) to develop the tFNA-Typ complex (TTC) for targeting mitochondria and treating AKI, which managed to restore mitochondrial function [154]. Nanotechnology-mediated antioxidative therapy is another target of research in AKI. Curcumin-loaded nanodrug delivery system (NPS@Cur) was studied to assess its effects on apoptosis, autophagy, and ER stress in AKI [155]. The results from cisplatin-induced AKI models revealed that NPS@Cur alleviates mitochondrial injury, which subsequently leads to kidney protection through antioxidative protection, regulated autophagy, and reduced ER stress. In addition, adenosine and related purinergic molecules, which play central roles in energy metabolism, have recently been implicated in the regulation of mitochondrial function in renal cells and may represent novel therapeutic avenues [155].

## 8. Future Perspective

Future research on mitochondrial dysfunction in kidney disease holds promise for significant advances in diagnosis, prevention, and treatment. Mitochondrial transplantation represents a groundbreaking therapeutic approach, with preliminary studies demonstrating its ability to mitigate acute kidney injury by modulating inflammatory pathways and enhancing bioenergetics [156]. Novel nanomedicine approaches, including the COPT nanoparticles that induce BNIP3-mediated mitophagy and curcumin-loaded delivery systems, show promise in targeting mitochondrial pathology with high specificity and reduced systemic toxicity [133]. Mitochondrial biomarkers, particularly urinary mitochondrial DNA, hold promise for the early detection and monitoring of disease progression, with research suggesting a correlation with declining eGFR and interstitial fibrosis in diabetic nephropathy [157,158,159,160]. The field is moving toward personalized approaches, where treatment strategies target specific mitochondrial pathways based on individual patient profiles, including genetic background, disease etiology, and hormonal status. Epigenetic interventions, such as PPAR-α agonists and KLF4 modulators, represent another frontier in personalizing mitochondrial-targeted therapies. Translational research, which bridges preclinical discoveries to clinical applications, is essential, with emerging technologies like tFNA delivery systems progressing toward human trials. Integrating multi-omics approaches with traditional clinical parameters will likely enhance our ability to stratify patients and predict responses to mitochondrial-targeted interventions, ultimately improving outcomes in the diverse spectrum of kidney diseases where mitochondrial dysfunction plays a central role.

## 9. Conclusions

This review highlights the essential role of mitochondria in kidney health and disease. Mitochondria are key players in energy production and maintaining cellular balance, and they also interact intricately with various signaling pathways and epigenetic mechanisms. Their involvement is particularly significant in the development of acute kidney injury (AKI), chronic kidney disease (CKD), and congenital anomalies of the kidney and urinary tract (CAKUT). Mitochondrial dysfunction, characterized by impaired dynamics, increased production of reactive oxygen species (ROS), and disrupted metabolic processes, is a significant contributor to renal inflammation, fibrosis, and structural abnormalities. Therefore, targeting mitochondrial function is a promising therapeutic approach to combat kidney disease and improve patient outcomes.

## Figures and Tables

**Table 1:** Targeting mitophagy and mitophagy-related signaling in kidney disease.

Treatment	Type	Stage	Target Pathway	Key Indication	Reference
TJ0113	Small molecule	Phase II	PINK1/Parkin activation	AS	[137]
Magnolol	Natural metabolite	Experimental	FUNDC1/BNIP3 activation	CKD fibrosis	[138]
Huangkui Capsule	Herbal formulation	Phase IV	STING1/PINK1 axis	DCKD	[139]
Astragaloside IV	Botanical extract	Experimental	PINK1/Parkin modulation	DCKD-TI	[140]
Metformin	AMPK agonist	Off-label use	AMPK/PINK1/Parkin	DCKD	[141]
LY344864	5-HT1F receptor agonist	Preclinical	Mitochondrial biogenesis	AKI	[142]
MitoQ	Synthetic antioxidant	Phase II	mtROS	DCKD/HCKD	[143]
Elamipretide	Mitochondrial peptide	Phase II	Mitochondrial cristae	DCKD	[144]
Urolithin A	Natural metabolite	Experimental	Mitophagy in tubular cells	HN	[145]
Pirfenidone	Anti-fibrotic	Phase II	Reduces kidney fibrosis	DCKD	[146]
Selonsertib	ASK1 inhibitor	Phase II	JNK pathway	DCKD	[147]
Esculetin	Coumarin derivative	Experimental	PINK1/Parkin mitophagy	DOX-induced KI	[148]
Lademirsen	miR-21 antagonist	Phase II	Inflammatory pathways	AS	[149]

Abbreviations: AMPK, AMP-activated protein kinase; 5-HT1F, 5-hydroxytryptamine; ASK1, Apoptosis signal-regulating kinase 1; PINK1, PTEN-induced kinase 1; FUNDC1, FUN14 domain-containing 1; BNIP3, BCL2 Interacting Protein 3; STING, Stimulator of Interferon Genes; mtROS; mitochondrial reactive oxygen species; JNK, Jun kinase; AS, Alport syndrome; CKD, chronic kidney disease; DCKD, diabetic chronic kidney disease; HCKD, hypertensive chronic kidney disease; TI, tubulointerstitial injury; AKI, acute kidney injury; HN, hyperuricemic nephropathy; DOX, doxorubicine.

## Data Availability

Not applicable.
